# {6,6′-Dimeth­oxy-2,2′-[4-bromo-*o*-phenyl­enebis(nitrilo­methyl­idyne)]diphenolato}nickel(II) methanol solvate

**DOI:** 10.1107/S1600536809002104

**Published:** 2009-01-23

**Authors:** Ming-Ming Yu, Hong Xu, Qiu-Zhi Shi, Ying-Nai Wei, Zhan-Xian Li

**Affiliations:** a Department of Chemistry, Zhengzhou University, Zhengzhou 450001, People’s Republic of China; b College of Physical Science and Engineering, Zhengzhou University, Zhengzhou 450001, People’s Republic of China

## Abstract

In the title compound, [Ni(C_22_H_17_BrN_2_O_4_)]·CH_3_OH, the Ni^II^ ion is in a slightly distorted square-planar geometry involving an N_2_O_2_ atom set of the tetra­dentate Schiff base ligand. The asymmetric unit contains one nickel complex and one methanol solvent mol­ecule. The dihedral angle between the aromatic ring planes of the central aromatic ring and other two aromatic rings are 10.8 (3) and 6.0 (2)°. The crystal structure is stabilized by inter­molecular C—H⋯O and C—H⋯Br and by intra­molecular O—H⋯O hydrogen bonds.

## Related literature

For Schiff base complexes in coordination chemistry, inorganic biochemistry, catalysis and optical materials, see: Aurangzeb *et al.* (1994 ▶); Fun & Kia (2008 ▶); Hulme *et al.* (1997 ▶); Li *et al.* (2008 ▶); Fei & Fang (2008 ▶); Xia *et al.* (2007 ▶); Zhang & Janiak (2001 ▶).
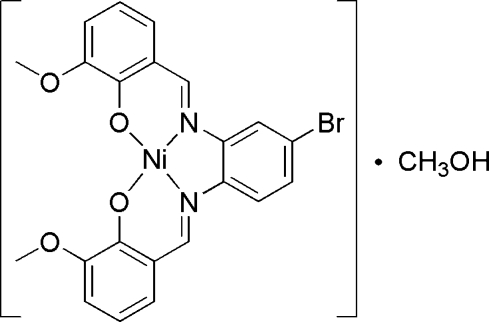


## Experimental

### 

#### Crystal data

[Ni(C_22_H_17_BrN_2_O_4_)]·CH_4_O
*M* *_r_* = 544.04
Triclinic, 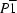

*a* = 7.4991 (12) Å
*b* = 11.8367 (18) Å
*c* = 12.5428 (19) Å
α = 105.042 (2)°
β = 96.971 (3)°
γ = 95.932 (3)°
*V* = 1056.6 (3) Å^3^
*Z* = 2
Mo *K*α radiation
μ = 2.85 mm^−1^
*T* = 295 (2) K
0.12 × 0.08 × 0.04 mm

#### Data collection

Bruker SMART 1K CCD area-detector diffractometer
Absorption correction: multi-scan (*SADABS*; Bruker, 2000 ▶) *T*_min_ = 0.726, *T*_max_ = 0.895
5680 measured reflections
4086 independent reflections
3123 reflections with *I* > 2σ(*I*)
*R*_int_ = 0.018

#### Refinement

*R*[*F* ^2^ > 2σ(*F* ^2^)] = 0.044
*wR*(*F* ^2^) = 0.161
*S* = 1.14
4086 reflections
291 parameters
H-atom parameters constrained
Δρ_max_ = 0.67 e Å^−3^
Δρ_min_ = −0.61 e Å^−3^

### 

Data collection: *SMART* (Bruker, 2000 ▶); cell refinement: *SAINT* (Bruker, 2000 ▶); data reduction: *SAINT*; program(s) used to solve structure: *SHELXTL* (Sheldrick, 2008 ▶); program(s) used to refine structure: *SHELXTL*; molecular graphics: *SHELXTL* software used to prepare material for publication: *SHELXTL*.

## Supplementary Material

Crystal structure: contains datablocks I, global. DOI: 10.1107/S1600536809002104/hg2467sup1.cif

Structure factors: contains datablocks I. DOI: 10.1107/S1600536809002104/hg2467Isup2.hkl

Additional supplementary materials:  crystallographic information; 3D view; checkCIF report

## Figures and Tables

**Table:** 

N2—Ni1	1.862 (3)
N3—Ni1	1.851 (3)
O1—Ni1	1.841 (3)
O2—Ni1	1.840 (3)

**Table:** 

O2—Ni1—O1	84.82 (12)
O2—Ni1—N3	93.97 (14)
O1—Ni1—N2	94.73 (14)
N3—Ni1—N2	86.59 (15)

**Table 2:** Hydrogen-bond geometry (Å, °)

*D*—H⋯*A*	*D*—H	H⋯*A*	*D*⋯*A*	*D*—H⋯*A*
O5—H5′⋯O1	0.82	2.15	2.952 (4)	165
C15—H15⋯O5^i^	0.93	2.37	3.203 (5)	149
C23—H23*B*⋯Br1^ii^	0.96	2.84	3.556 (6)	132

Symmetry codes: (i) 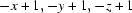; (ii) 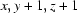.

**Fig. 1.**
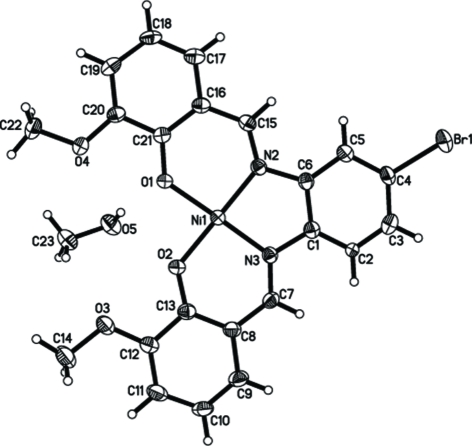
A view of complex (I), showing 30% probability displacement ellipsoids and the atom-numbering scheme.

**Fig. 2.**
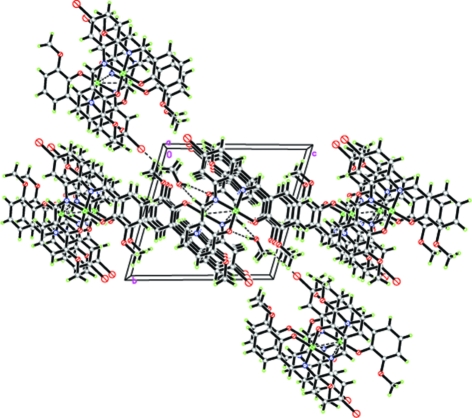
The crystal packing of title compound, viewed along the a axis. Hydrogen bonds are shown as dashed lines.

## supplementary crystallographic information

### Comment

The synthesis and structural investigation of Schiff base complexes have attracted much attention due to their interesting structures and wide potential applications. They play an important role in the development of coordination chemistry as well as inorganic biochemistry, catalysis and optical materials (Aurangzeb *et al.*, 1994; Fun & Kia, 2008; Hulme *et al.*, 1997; Li *et al.*, 2008; Fei & Fang, 2008; Zhang & Janiak, 2001). Here, the synthesis and crystal structure of the title complex (I) are reported.

The molecular structure of title compound is showing in Fig. 1. The dihedral angles between the aromatic ring planes of the middle aromatic ring and other two aromatic rings are 10.8 (3)° and 6.0 (2)°, respectively. The crystal structure, is stabilized by intermolecular C—H···O and C—H···Br and intramolecular O—H···O hydrogen bonds.

### Experimental

6,6'-Dimethoxy-2,2'-[-4-bromo-*o*-phenylenebis (nitrilomethylidyne)]diphenol was synthesized according to modified reported methods (Xia, *et al.*, 2007). A mixture of NiCl_2_.6H_2_O (1 mmol, 237.7 mg), 6,6'-Dimethoxy-2,2'-[-4-bromo-*o*-phenylenebis (nitrilomethylidyne)]diphenol (1 mmol, 455.3 mg) in 40 ml methanol and 20 ml water was refluxed for forty minutes. Crystals suitable for X-ray diffraction analysis were obtained by slow evaporation at room temperature for three weeks.

### Refinement

All H atoms were placed in geometrically calculated positions with C—H = 0.96 Å for methyl H atoms, C—H = 0.93 Å for aromatic H atoms and were refined isotropic with *U*_iso_(H) = 1.2*U*_eq_(C) of parent atom using a riding model. H atoms of methanol were constrained to idealized geometries, with C—H = 0.96 Å for methyl H atoms, O—H = 0.82 Å, and with *U*_iso_(H) = 1.2*U*_eq_(C) and 1.5*U*_eq_(O).

### Crystal data

**Table:**

[Ni(C_22_H_17_BrN_2_O_4_)]·CH_4_O	*Z* = 2
*M**_r_* = 544.04	*F*(000) = 552
Triclinic, *P*1	*D*_x_ = 1.710 Mg m^−^^3^
Hall symbol: -P 1	Mo *K*α radiation, λ = 0.71073 Å
*a* = 7.4991 (12) Å	Cell parameters from 4086 reflections
*b* = 11.8367 (18) Å	θ = 1.7–26.0°
*c* = 12.5428 (19) Å	µ = 2.85 mm^−^^1^
α = 105.042 (2)°	*T* = 295 K
β = 96.971 (3)°	Block, brown
γ = 95.932 (3)°	0.12 × 0.08 × 0.04 mm
*V* = 1056.6 (3) Å^3^	

### Data collection

**Table:**

Bruker SMART 1K CCD area-detector diffractometer	4086 independent reflections
Radiation source: fine-focus sealed tube	3123 reflections with *I* > 2σ(*I*)
graphite	*R*_int_ = 0.018
φ and ω scans	θ_max_ = 26.0°, θ_min_ = 1.7°
Absorption correction: multi-scan (*SADABS*; Bruker, 2000)	*h* = −9→9
*T*_min_ = 0.726, *T*_max_ = 0.895	*k* = −14→12
5680 measured reflections	*l* = −13→15

### Refinement

**Table:**

Refinement on *F*^2^	Primary atom site location: structure-invariant direct methods
Least-squares matrix: full	Secondary atom site location: difference Fourier map
*R*[*F*^2^ > 2σ(*F*^2^)] = 0.044	Hydrogen site location: inferred from neighbouring sites
*wR*(*F*^2^) = 0.161	H-atom parameters constrained
*S* = 1.14	*w* = 1/[σ^2^(*F*_o_^2^) + (0.0951*P*)^2^ + 0.0167*P*] where *P* = (*F*_o_^2^ + 2*F*_c_^2^)/3
4086 reflections	(Δ/σ)_max_ = 0.003
291 parameters	Δρ_max_ = 0.67 e Å^−^^3^
0 restraints	Δρ_min_ = −0.61 e Å^−^^3^

### Special details

**Table:**

Geometry. All e.s.d.'s (except the e.s.d. in the dihedral angle between two l.s. planes) are estimated using the full covariance matrix. The cell e.s.d.'s are taken into account individually in the estimation of e.s.d.'s in distances, angles and torsion angles; correlations between e.s.d.'s in cell parameters are only used when they are defined by crystal symmetry. An approximate (isotropic) treatment of cell e.s.d.'s is used for estimating e.s.d.'s involving l.s. planes.
Refinement. Refinement of *F*^2^ against ALL reflections. The weighted *R*-factor *wR* and goodness of fit *S* are based on *F*^2^, conventional *R*-factors *R* are based on *F*, with *F* set to zero for negative *F*^2^. The threshold expression of *F*^2^ > σ(*F*^2^) is used only for calculating *R*-factors(gt) *etc*. and is not relevant to the choice of reflections for refinement. *R*-factors based on *F*^2^ are statistically about twice as large as those based on *F*, and *R*- factors based on ALL data will be even larger.

### Fractional atomic coordinates and isotropic or equivalent isotropic displacement parameters (Å^2^)

**Table:**

	*x*	*y*	*z*	*U*_iso_*/*U*_eq_	
C3	0.8652 (7)	0.0691 (4)	0.3998 (4)	0.0510 (12)	
H3	0.8869	−0.0085	0.3912	0.061*	
C4	0.7842 (7)	0.1013 (4)	0.3070 (4)	0.0471 (11)	
C5	0.7428 (6)	0.2142 (4)	0.3178 (4)	0.0443 (10)	
H5	0.6876	0.2363	0.2573	0.053*	
C6	0.7873 (5)	0.2926 (4)	0.4224 (3)	0.0357 (9)	
C1	0.8774 (6)	0.2549 (4)	0.5093 (3)	0.0370 (9)	
C7	1.0169 (6)	0.3214 (4)	0.6961 (4)	0.0393 (10)	
H7	1.0357	0.2435	0.6888	0.047*	
C8	1.0843 (6)	0.4041 (4)	0.7991 (4)	0.0413 (10)	
C9	1.1868 (7)	0.3673 (5)	0.8839 (4)	0.0521 (12)	
H9	1.2006	0.2879	0.8717	0.063*	
C10	1.2639 (7)	0.4443 (5)	0.9811 (4)	0.0569 (13)	
H10	1.3277	0.4176	1.0358	0.068*	
C11	1.2491 (7)	0.5644 (5)	1.0008 (4)	0.0552 (13)	
H11	1.3035	0.6174	1.0683	0.066*	
C12	1.1539 (7)	0.6045 (4)	0.9200 (4)	0.0477 (11)	
C13	1.0650 (6)	0.5251 (4)	0.8169 (3)	0.0397 (10)	
C14	1.2135 (11)	0.8037 (5)	1.0330 (5)	0.093 (2)	
H14B	1.3419	0.8015	1.0451	0.139*	
H14A	1.1605	0.7842	1.0930	0.139*	
H14C	1.1918	0.8815	1.0304	0.139*	
C15	0.6634 (5)	0.4582 (4)	0.3866 (3)	0.0368 (9)	
H15	0.6145	0.4089	0.3162	0.044*	
C16	0.6301 (5)	0.5774 (4)	0.4112 (3)	0.0371 (9)	
C17	0.5302 (6)	0.6144 (5)	0.3271 (4)	0.0489 (11)	
H17	0.4833	0.5601	0.2588	0.059*	
C18	0.5021 (7)	0.7289 (5)	0.3451 (4)	0.0517 (12)	
H18	0.4382	0.7527	0.2883	0.062*	
C19	0.5680 (6)	0.8118 (4)	0.4482 (4)	0.0503 (12)	
H19	0.5483	0.8900	0.4591	0.060*	
C20	0.6611 (6)	0.7783 (4)	0.5328 (4)	0.0395 (10)	
C21	0.6960 (5)	0.6590 (4)	0.5160 (3)	0.0343 (9)	
C22	0.7016 (8)	0.9703 (4)	0.6604 (4)	0.0581 (13)	
H22A	0.7580	1.0111	0.7354	0.087*	
H22B	0.5737	0.9744	0.6532	0.087*	
H22C	0.7536	1.0067	0.6091	0.087*	
C23	0.7099 (11)	0.8110 (6)	0.8879 (5)	0.087 (2)	
H23A	0.8401	0.8205	0.8984	0.131*	
H23B	0.6687	0.8131	0.9578	0.131*	
H23C	0.6685	0.8739	0.8607	0.131*	
C2	0.9109 (5)	0.1459 (3)	0.4982 (3)	0.0303 (8)	
H2	0.9658	0.1238	0.5588	0.036*	
N2	0.7574 (4)	0.4124 (3)	0.4553 (3)	0.0349 (8)	
N3	0.9301 (4)	0.3451 (3)	0.6101 (3)	0.0343 (7)	
O1	0.7893 (4)	0.6322 (2)	0.5995 (2)	0.0385 (7)	
O2	0.9756 (4)	0.5679 (3)	0.7446 (2)	0.0416 (7)	
O3	1.1343 (6)	0.7207 (3)	0.9303 (3)	0.0678 (11)	
O4	0.7307 (5)	0.8499 (3)	0.6360 (3)	0.0501 (8)	
O5	0.6410 (5)	0.7031 (3)	0.8107 (3)	0.0665 (10)	
H5'	0.6896	0.6966	0.7546	0.100*	
Ni1	0.86214 (7)	0.48899 (4)	0.60223 (4)	0.03314 (19)	
Br1	0.73229 (10)	−0.01370 (5)	0.16760 (5)	0.0796 (3)	

### Atomic displacement parameters (Å^2^)

**Table:**

	*U*^11^	*U*^22^	*U*^33^	*U*^12^	*U*^13^	*U*^23^
C3	0.053 (3)	0.038 (2)	0.064 (3)	0.013 (2)	0.013 (2)	0.013 (2)
C4	0.060 (3)	0.037 (2)	0.036 (2)	0.004 (2)	0.003 (2)	−0.0015 (19)
C5	0.051 (3)	0.042 (2)	0.036 (2)	0.006 (2)	0.004 (2)	0.0065 (19)
C6	0.034 (2)	0.033 (2)	0.037 (2)	0.0021 (16)	0.0070 (18)	0.0065 (18)
C1	0.033 (2)	0.038 (2)	0.039 (2)	0.0048 (17)	0.0081 (18)	0.0082 (19)
C7	0.038 (2)	0.040 (2)	0.044 (2)	0.0129 (18)	0.0070 (19)	0.0136 (19)
C8	0.039 (2)	0.049 (3)	0.036 (2)	0.0110 (19)	0.0031 (19)	0.013 (2)
C9	0.064 (3)	0.059 (3)	0.042 (3)	0.022 (2)	0.007 (2)	0.023 (2)
C10	0.059 (3)	0.069 (4)	0.045 (3)	0.023 (3)	−0.006 (2)	0.021 (3)
C11	0.052 (3)	0.066 (3)	0.040 (3)	0.004 (2)	−0.008 (2)	0.009 (2)
C12	0.052 (3)	0.049 (3)	0.039 (2)	0.006 (2)	−0.002 (2)	0.012 (2)
C13	0.039 (2)	0.046 (2)	0.037 (2)	0.0074 (18)	0.0071 (18)	0.0149 (19)
C14	0.146 (7)	0.053 (4)	0.055 (4)	−0.014 (4)	−0.031 (4)	0.006 (3)
C15	0.033 (2)	0.042 (2)	0.034 (2)	−0.0010 (17)	0.0043 (17)	0.0092 (18)
C16	0.029 (2)	0.046 (2)	0.038 (2)	0.0049 (17)	0.0049 (17)	0.0158 (19)
C17	0.044 (3)	0.062 (3)	0.043 (3)	0.009 (2)	−0.001 (2)	0.020 (2)
C18	0.049 (3)	0.066 (3)	0.046 (3)	0.018 (2)	−0.005 (2)	0.028 (2)
C19	0.049 (3)	0.048 (3)	0.063 (3)	0.015 (2)	0.008 (2)	0.029 (2)
C20	0.037 (2)	0.045 (2)	0.041 (2)	0.0072 (18)	0.0078 (19)	0.019 (2)
C21	0.028 (2)	0.040 (2)	0.037 (2)	0.0026 (16)	0.0065 (17)	0.0145 (18)
C22	0.074 (3)	0.036 (3)	0.066 (3)	0.018 (2)	0.012 (3)	0.014 (2)
C23	0.119 (6)	0.074 (4)	0.051 (3)	0.021 (4)	−0.015 (4)	−0.005 (3)
C2	0.034 (2)	0.0273 (19)	0.0283 (19)	0.0080 (15)	0.0012 (16)	0.0056 (16)
N2	0.0321 (17)	0.0362 (18)	0.0341 (18)	0.0026 (14)	0.0040 (15)	0.0070 (15)
N3	0.0323 (17)	0.0354 (18)	0.0341 (18)	0.0054 (14)	0.0072 (14)	0.0067 (15)
O1	0.0466 (17)	0.0355 (15)	0.0317 (15)	0.0087 (13)	−0.0023 (13)	0.0092 (12)
O2	0.0493 (17)	0.0393 (16)	0.0346 (15)	0.0074 (13)	−0.0034 (13)	0.0116 (13)
O3	0.100 (3)	0.044 (2)	0.045 (2)	−0.0022 (19)	−0.017 (2)	0.0069 (16)
O4	0.066 (2)	0.0355 (16)	0.0469 (19)	0.0123 (15)	0.0009 (16)	0.0103 (14)
O5	0.069 (3)	0.073 (3)	0.048 (2)	0.007 (2)	0.0067 (18)	0.0035 (18)
Ni1	0.0361 (3)	0.0314 (3)	0.0302 (3)	0.0051 (2)	0.0022 (2)	0.0068 (2)
Br1	0.1203 (6)	0.0507 (4)	0.0512 (4)	0.0185 (3)	−0.0019 (3)	−0.0107 (3)

### Geometric parameters (Å, °)

**Table:**

C3—C2	1.310 (6)	C15—N2	1.307 (5)
C3—C4	1.406 (7)	C15—C16	1.419 (6)
C3—H3	0.9300	C15—H15	0.9300
C4—C5	1.380 (7)	C16—C17	1.408 (6)
C4—Br1	1.885 (4)	C16—C21	1.411 (6)
C5—C6	1.376 (6)	C17—C18	1.358 (7)
C5—H5	0.9300	C17—H17	0.9300
C6—C1	1.406 (6)	C18—C19	1.401 (7)
C6—N2	1.419 (5)	C18—H18	0.9300
C1—C2	1.316 (6)	C19—C20	1.366 (6)
C1—N3	1.409 (5)	C19—H19	0.9300
C7—N3	1.301 (5)	C20—O4	1.357 (5)
C7—C8	1.402 (6)	C20—C21	1.429 (6)
C7—H7	0.9300	C21—O1	1.315 (5)
C8—C13	1.417 (6)	C22—O4	1.423 (5)
C8—C9	1.423 (6)	C22—H22A	0.9600
C9—C10	1.339 (7)	C22—H22B	0.9600
C9—H9	0.9300	C22—H22C	0.9600
C10—C11	1.397 (7)	C23—O5	1.392 (7)
C10—H10	0.9300	C23—H23A	0.9600
C11—C12	1.381 (6)	C23—H23B	0.9600
C11—H11	0.9300	C23—H23C	0.9600
C12—O3	1.373 (6)	C2—H2	0.9300
C12—C13	1.425 (6)	N2—Ni1	1.862 (3)
C13—O2	1.299 (5)	N3—Ni1	1.851 (3)
C14—O3	1.419 (6)	O1—Ni1	1.841 (3)
C14—H14B	0.9600	O2—Ni1	1.840 (3)
C14—H14A	0.9600	O5—H5'	0.8200
C14—H14C	0.9600		
			
C2—C3—C4	121.5 (4)	C18—C17—C16	120.4 (4)
C2—C3—H3	119.3	C18—C17—H17	119.8
C4—C3—H3	119.3	C16—C17—H17	119.8
C5—C4—C3	120.8 (4)	C17—C18—C19	120.9 (4)
C5—C4—Br1	120.8 (4)	C17—C18—H18	119.6
C3—C4—Br1	118.4 (4)	C19—C18—H18	119.6
C6—C5—C4	116.7 (4)	C20—C19—C18	120.4 (4)
C6—C5—H5	121.6	C20—C19—H19	119.8
C4—C5—H5	121.6	C18—C19—H19	119.8
C5—C6—C1	118.7 (4)	O4—C20—C19	125.8 (4)
C5—C6—N2	127.6 (4)	O4—C20—C21	114.0 (4)
C1—C6—N2	113.7 (3)	C19—C20—C21	120.2 (4)
C2—C1—C6	123.6 (4)	O1—C21—C16	123.9 (4)
C2—C1—N3	122.6 (4)	O1—C21—C20	117.6 (4)
C6—C1—N3	113.9 (4)	C16—C21—C20	118.5 (4)
N3—C7—C8	125.1 (4)	O4—C22—H22A	109.5
N3—C7—H7	117.4	O4—C22—H22B	109.5
C8—C7—H7	117.4	H22A—C22—H22B	109.5
C7—C8—C13	121.2 (4)	O4—C22—H22C	109.5
C7—C8—C9	119.1 (4)	H22A—C22—H22C	109.5
C13—C8—C9	119.5 (4)	H22B—C22—H22C	109.5
C10—C9—C8	121.5 (5)	O5—C23—H23A	109.5
C10—C9—H9	119.3	O5—C23—H23B	109.5
C8—C9—H9	119.3	H23A—C23—H23B	109.5
C9—C10—C11	120.5 (4)	O5—C23—H23C	109.5
C9—C10—H10	119.7	H23A—C23—H23C	109.5
C11—C10—H10	119.7	H23B—C23—H23C	109.5
C12—C11—C10	120.0 (5)	C3—C2—C1	118.5 (4)
C12—C11—H11	120.0	C3—C2—H2	120.7
C10—C11—H11	120.0	C1—C2—H2	120.7
O3—C12—C11	124.5 (4)	C15—N2—C6	121.1 (3)
O3—C12—C13	114.2 (4)	C15—N2—Ni1	126.4 (3)
C11—C12—C13	121.3 (5)	C6—N2—Ni1	112.5 (3)
O2—C13—C8	124.3 (4)	C7—N3—C1	119.6 (4)
O2—C13—C12	118.5 (4)	C7—N3—Ni1	127.2 (3)
C8—C13—C12	117.2 (4)	C1—N3—Ni1	113.2 (3)
O3—C14—H14B	109.5	C21—O1—Ni1	127.9 (3)
O3—C14—H14A	109.5	C13—O2—Ni1	128.0 (3)
H14B—C14—H14A	109.5	C12—O3—C14	116.9 (4)
O3—C14—H14C	109.5	C20—O4—C22	117.9 (4)
H14B—C14—H14C	109.5	C23—O5—H5'	109.5
H14A—C14—H14C	109.5	O2—Ni1—O1	84.82 (12)
N2—C15—C16	125.1 (4)	O2—Ni1—N3	93.97 (14)
N2—C15—H15	117.5	O1—Ni1—N3	177.54 (13)
C16—C15—H15	117.5	O2—Ni1—N2	177.07 (14)
C17—C16—C21	119.7 (4)	O1—Ni1—N2	94.73 (14)
C17—C16—C15	118.3 (4)	N3—Ni1—N2	86.59 (15)
C21—C16—C15	122.0 (4)		

### Hydrogen-bond geometry (Å, °)

**Table:**

*D*—H···*A*	*D*—H	H···*A*	*D*···*A*	*D*—H···*A*
O5—H5'···O1	0.82	2.15	2.952 (4)	165
C15—H15···O5^i^	0.93	2.37	3.203 (5)	149
C23—H23B···Br1^ii^	0.96	2.84	3.556 (6)	133

Symmetry codes: (i) −*x*+1, −*y*+1, −*z*+1; (ii) *x*, *y*+1, *z*+1.
